# Bringing nature indoors: characterizing the unique contribution of fractal structure and the effects of Euclidean context on perception of fractal patterns

**DOI:** 10.3389/fpsyg.2023.1210584

**Published:** 2023-08-24

**Authors:** Kelly E. Robles, Nate Gonzales-Hess, Richard P. Taylor, Margaret E. Sereno

**Affiliations:** ^1^Integrative Perception Lab, Psychology Department, University of Oregon, Eugene, OR, United States; ^2^Material Science Institute, Physics Department, University of Oregon, Eugene, OR, United States

**Keywords:** fractal design, composite fractals, fractal dimension, preference, aesthetics

## Abstract

Imbuing the benefits of natural design into humanmade spaces, installations of fractal patterns have been employed to shape occupant experience. Previous work has demonstrated consistent trends for fractal judgments in the presence of design elements. The current study identifies the extent to which underlying pattern structure and perceptions of pattern complexity drive viewer judgments, and how response trends are altered with the incorporation of Euclidean context reminiscent of indoor spaces. This series of studies first establishes that pattern appeal, interest, naturalness, and relaxation have a fundamentally inverse relationship with perceptions of pattern complexity and that the presence of fractal structure contributes uniquely and positively to pattern perception. Subsequently, the addition of Euclidean structure establishes a discrete pattern boundary that alters fractal perceptions of interest and excitement but not the remaining judgments. The presence of consistent subpopulations, particularly those that contradict overarching perceptual trends is supported across studies, and further emphasizes the importance of adjusting pattern selection to consider the greatest number of possible viewers. Through informed pattern selection, designs can be installed to maximize desired experience of a space while minimizing negative impressions bound to arise in a minority of occupants. This set of studies demonstrates that through control of perceived pattern complexity and whether an emphasis is placed on pattern boundaries, fractal patterns can serve to establish predictable experiences of humanmade spaces in order to inject the benefits of nature into manufactured environments.

## Introduction

The positive impact of bringing nature indoors reaches far beyond the esthetic benefits of adding a plant to an office windowsill (Berman et al., 2008; Korpela et al., 2017). Fractals embody the self-similar pattern repetition found throughout nature (Mandelbrot, 1982; Taylor, 2021) and have been harnessed to improve occupant wellbeing (Taylor and Sprott, 2008; Smith et al., 2020). Exemplified by research supporting fractal fluency theory (Taylor and Spehar, 2016; Taylor et al., 2018) the visual system is tuned to more efficiently process fractal patterns of low-moderate complexity which are prevalent through nature (Spehar et al., 2003; Hagerhall et al., 2008; Taylor et al., 2018). This processing fluency supports perceptions of high esthetic quality (Friedenberg et al., 2021) as well as peaks in task performance (Ferreira et al., 2012; Spehar et al., 2015; Juliani et al., 2016; Taylor et al., 2017; Burtan et al., 2021). In the same manner in which exposure to nature encourages positive psychological states (Ulrich, 1981; Kaplan and Kaplan, 1982; Kaplan and Kaplan, 1989; Ulrich et al., 1991; Kellert, 1993; Hagerhall et al., 2015) incorporation of fractal patterns into visual surroundings supports the biophilic hypothesis of a fundamental human need for connection to nature (Wilson, 1984).

Across a robust body of research (Spehar et al., 2003; Bies et al., 2016a; Robles et al., 2020) as well as prevalence in artistic works (Taylor et al., 1999, 2007, 2018; Taylor, 2003; Graham and Field, 2008; Graham and Redies, 2010; Viengkham and Spehar, 2018), fractal arrangements have demonstrated a high esthetic quality. Judgments of fractal preference closely follow variations in perceived pattern complexity, with those possessing exact repetition and symmetry encouraging a greater tolerance for objective complexity than those that repeat in a statistical manner more common throughout the natural world (Taylor et al., 2005; Taylor and Sprott, 2008; Taylor et al., 2011; Hagerhall et al., 2015; Bies et al., 2016a; Robles et al., 2020). Objective pattern complexity results from variations in the relative coarse-to-fine pattern structure determined by internal pattern factors such as variations in recursion (number of repetitions across scales) and complexity of fractal dimension “*D*-value” (the rate of pattern shrinkage between repetitions to quantify the ratio of fine structure). Perceived pattern complexity also constrains broader pattern judgments (Abboushi et al., 2019; Robles et al., 2021). Selection of optimal fractal features expands beyond observed improvements to esthetic experiences of a given object, to facilitate viewer cognition and performance on a wide span of tasks (Juliani et al., 2016; Taylor et al., 2018; Abboushi et al., 2019; Roe et al., 2020; Spehar and Stevanov, 2021).

In stark contrast to the statistical configuration of nature embodied by fractals, humanmade spaces are composed of Euclidean arrangements which require further energy to sufficiently process (Taylor, 2006; Hagerhall et al., 2008; Le et al., 2017). Alongside diminished esthetic experiences (for review see Brielmann et al., 2022) greater time spent inside artificial environments coincides with negative health effects including visual strain, headaches, and general increases in feelings of stress (O’Hare and Hibbard, 2011; Penacchio and Wilkins, 2015; Ogawa and Motoyoshi, 2020). In recent years interior design has sought to inject more naturalistic elements into interior space to improve occupant experiences (Korpela et al., 2017; Smith et al., 2020). Specifically, fractal installations have been manufactured to explicitly address the problems of an overabundance of unnatural spatial frequencies in occupant space by providing an opportunity to help mitigate the negative effects of Euclidean environments without compromising the utility of a given structure (Taylor and Sprott, 2008; Roe et al., 2020; Smith et al., 2020). The benefit of bringing natural design indoors can be maximized through conscientious selection of fractal configurations that balance esthetic and perceptual properties of the pattern along with the confining factors of the space.

The current study investigates the influence of pattern structure on observer experiences, specifically how internal composition (with the presence or absence of fractal organization) and external context (with the presence or absence of surrounding Euclidean configuration) alter well-established trends in fractal perception (Abboushi et al., 2019; Robles et al., 2021). In the first ever study to compare ratings of fractal images to corresponding statistically matched nonfractal patterns, unipolar ratings are collected across a broad range of experiential measurements to isolate the impact of fractal structure on predictable viewer experience. The goal of this series of experiments is to establish an empirical basis for guiding optimal installment of fractal-based designs to maximize pattern effectiveness in eliciting various psychological experiences of a space. Moreover, results from subgroup analyses will inform calculated selection of designs that balance various internal pattern factors (including complexity and arrangement) as well as surrounding structure in order to accommodate occupants with contradictory preferences.

## Experiment 1—isolating the impact of fractal structure on pattern perception

### Materials and methods

#### Stimuli

**Figure 1:**
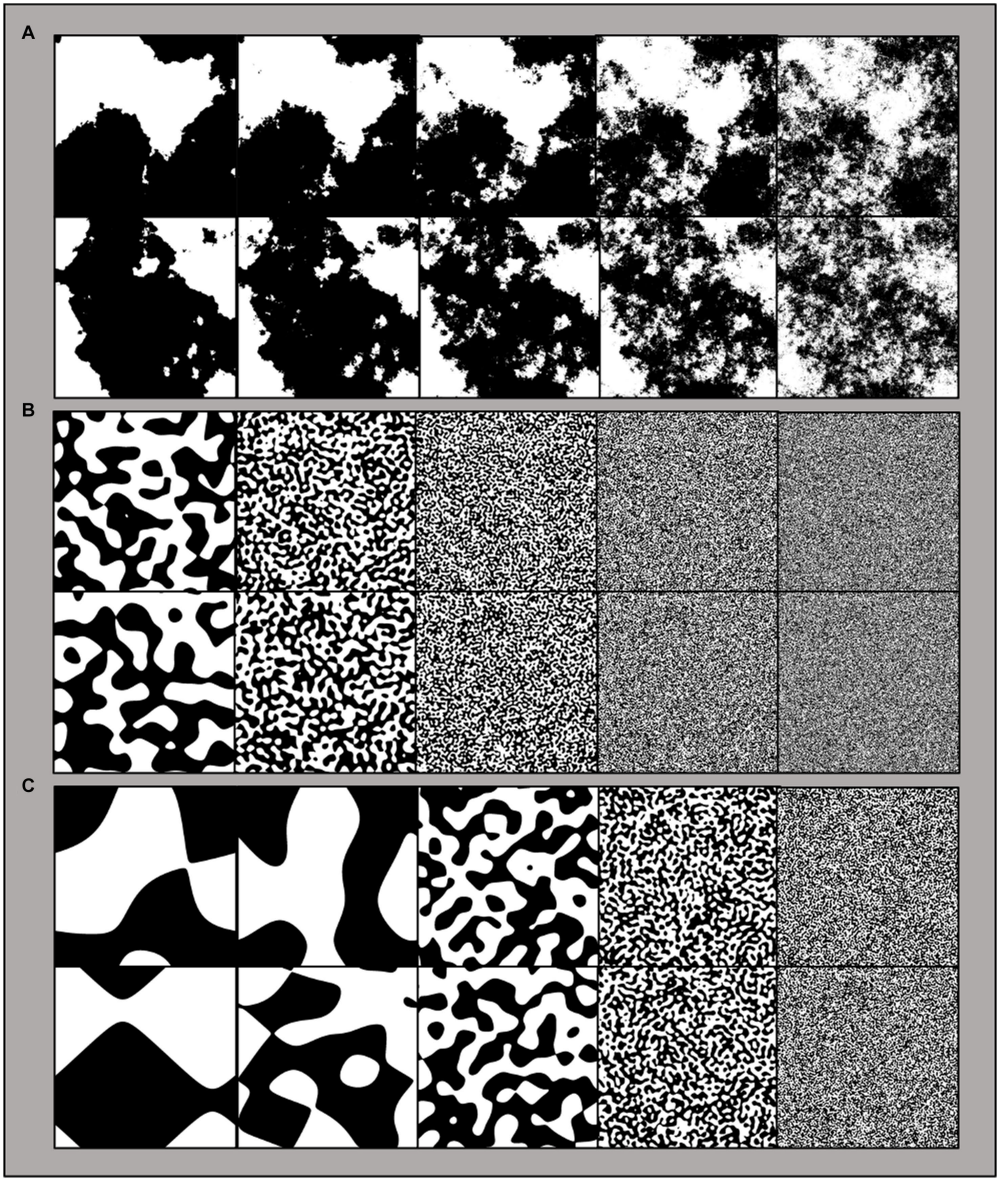
Examples of the five stimulus patterns generated by the same seed pattern and ranging from low (left column) to high complexity (right column). **(A)** Fractals: fractal patterns with a fractal dimension (*D*) = 1.1, 1.3, 1.5, 1.7, and 1.9. **(B)** Average-nonfractals: non-fractal patterns with region sizes averaged to match the average region size of the original fractal pattern. **(C)** Large-nonfractals: non-fractal patterns with region sizes averaged to match the large-scale region size of the original fractal pattern.

**Figure 2:**
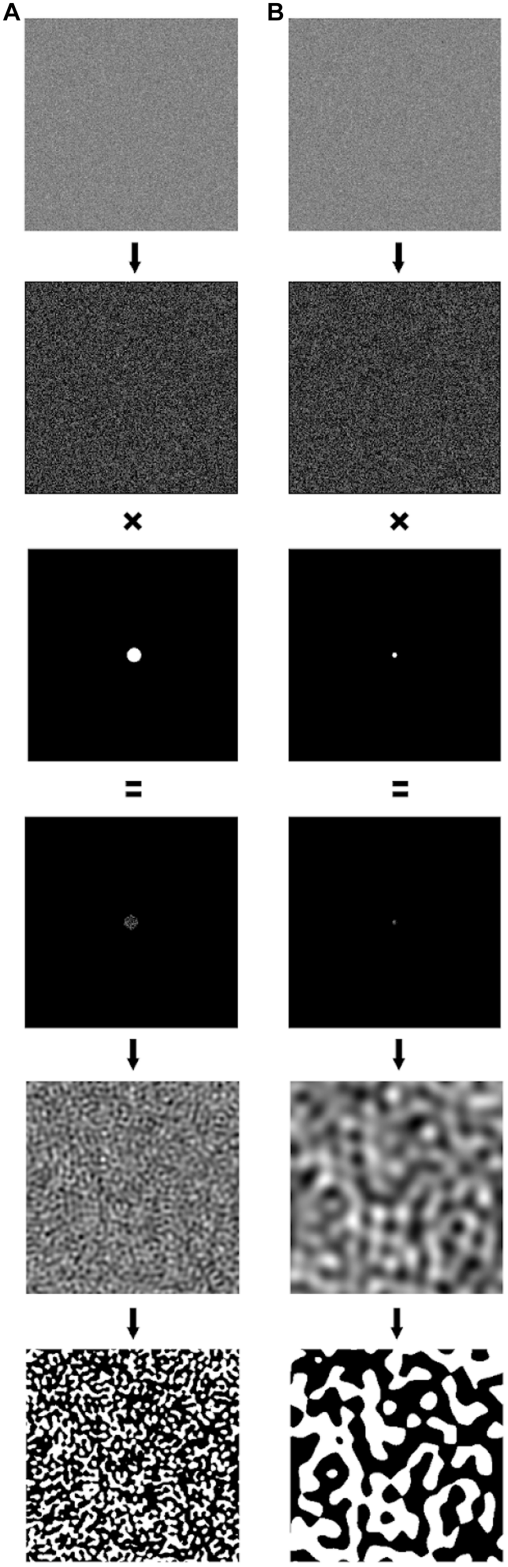
Two examples of the control stimulus generation process. A field of normally distributed white noise was generated, then processed using a Fast Fourier Transform to yield a 2-dimensional matrix representing the frequency domain. This matrix was then multiplied with a circular filter to eliminate some amount of high frequency information. Low frequency information was located in the center of the matrix, so a narrow filter eliminates all but the lowest frequencies, whereas a wider filter retains more high frequencies. The filtered matrix was then inverse Fast Fourier Transformed to yield the final noise pattern, which was then thresholded to produce a black and white image. Panel **(A)** shows a wide filter, which results in a large range of frequencies in the final image, Panel **(B)** employs a tighter filter, which eliminates higher frequencies and produces a final pattern with lower frequency information. The algorithm used to generate control stimuli systematically manipulated filter size to produce control stimuli that closely matched the average region size of a given fractal stimulus.

In order to isolate the unique influence of fractal structure, participants were presented with both fractal and matched non-fractal images. All stimuli are first generated as fractal images and then systematically altered to produce non-fractal matches. Fractal patterns are initially generated in a graphic user interface (GUI) using the midpoint displacement method to produce a series of 5 black-white fractal images per unique seed pattern (*D*-value of 1.1, 1.3, 1.5, 1.7, 1.9; see Bies et al., 2016b for generation specifics; Figure 1A). The non-fractal control stimuli were created by generating normally distributed white noise, which was then Fourier transformed and band-pass filtered to match the average region size of a given fractal stimulus (Figure 2). The control stimuli are matched to the 5 levels of fractal dimension in two ways: (1) by equating mean region size (mean number of contiguous white or black pixels > 3 pixels in a given fractal stimulus; “Average” control; see Figure 1B), and (2) by capturing larger scale structure by matching the upper mean (the mean of all above-average region sizes) in a given fractal image; “Large” scale control (see Figure 1C). This procedure generates control stimuli with similar levels of complexity to their fractal matches, but without their fractal characteristics.

#### Participants

To quantify the unique impact of underlying fractal structure on observer perceptions, 110 undergraduate Psychology students from the University of Oregon were recruited for the current study through the SONA participant pool system (69 females, age ranging between 18 and 32 years old, mean age 21 years old). Prior to participation, informed consent was acquired following a protocol approved by the Institutional Review Board at the University of Oregon and demographic information was collected. All participants were compensated with class credit.

#### Visual displays

Experiment 1 was generated in PsychoPy3 (Peirce et al., 2019). Participants were seated approximately 70 cm from a computer with 27-inch monitor with screen resolution of 2,560 × 1,440 pixels and 60 Hz refresh rate. Thus, stimuli size was roughly 720 × 720 pixels or 13.8 degrees of visual angle across.

#### Design and procedure

Participants completed 18 randomized blocks of self-report slider judgments, with each block consisting of one pattern type (fractal, Average-nonfractal, Large-nonfractal) and a singular judgment type (complexity, appeal, naturalness, interest, relaxing, exciting). Each block’s stimulus set consisted of 4 unique patterns ranging across 5 levels of complexity (equal or matched to a *D*-value 1.1, 1.3, 1.5, 1.7, 1.9) giving rise to 20 trials per block and 360 total stimulus-related trials across the experiment. At the start of each block, participants were instructed to make a single randomly ordered judgment for each stimulus presented in that block. Specifically, they were asked to answer one of 6 questions for each block: “How _______ is the image?” with one of 6 different words placed in the blank (complex, appealing, natural, interesting, relaxing, exciting). Participants rated each pattern by clicking on a slider located beneath the image ranging between 0 and 1, with the “0” end of the slider indicating “not at all” and the “1” end of the slider indicating “completely.” They were instructed to use the full range of the slider and stimuli remained on the screen until a response was recorded. Upon completion of the experiment, participants were debriefed according to the protocol approved by the Institutional Review Board at the University of Oregon.

### Results

Data from 94 participants were retained from the 110 individuals who participated in the experiment. Data were excluded due to (1) failure to complete the study (3 participants), (2) failure to follow directions (8 participants), or (3) in greater than 3 blocks, a participant made 4 or more consecutive ratings that were within a thousandth of a degree of one another (5 participants).

#### Pattern judgment task

**Figure 3:**
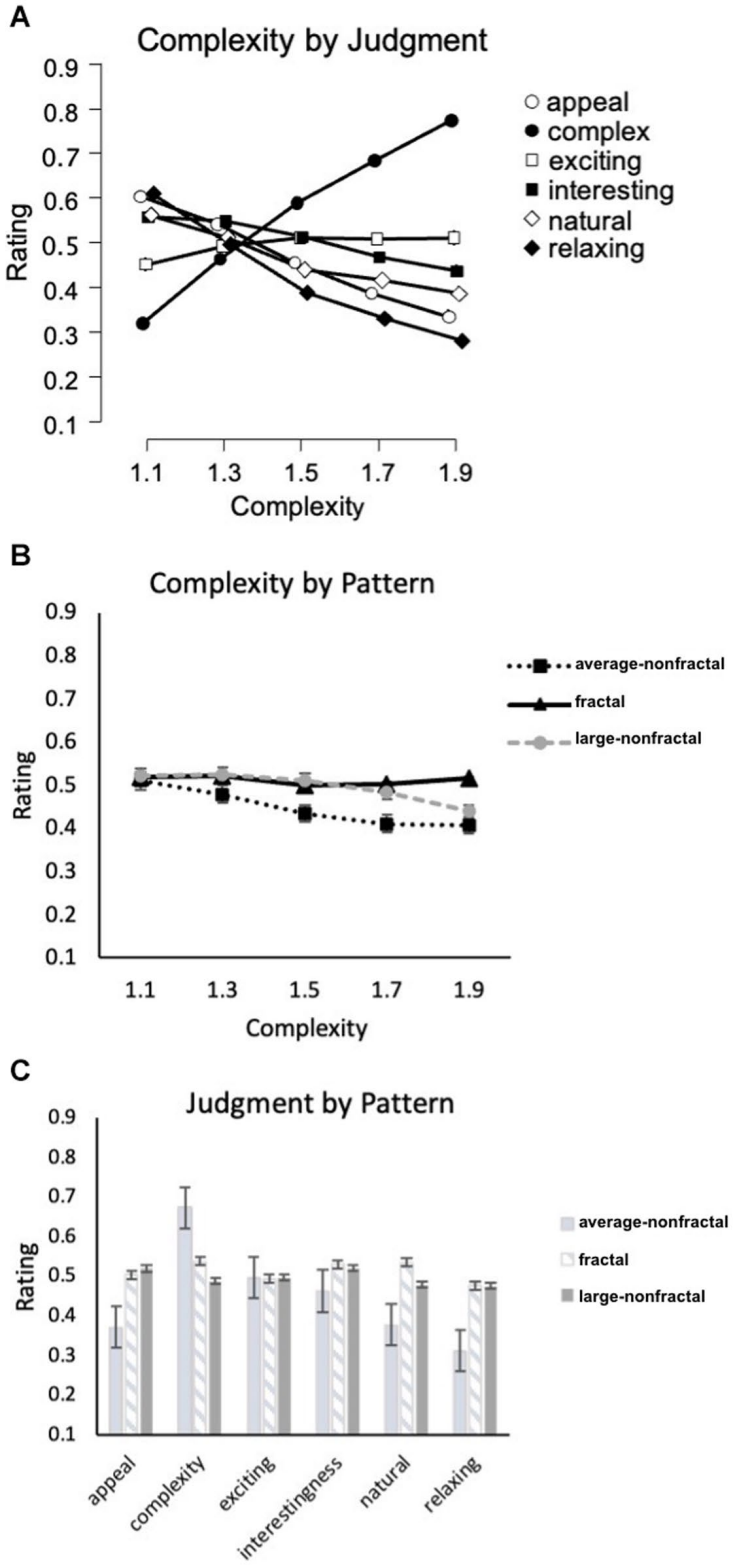
Experiment 1 results for the 3 pattern-types using a unipolar rating scale. Results show significant interactions among the experiment’s 3 factors: complexity, pattern type (fractal, Average-nonfractal, Large-nonfractal), and judgment type (appeal, complexity, exciting, interestingness, engaging, relaxing). Participant rating (on a scale from 0–1) is plotted as a function of **(A)** complexity and different judgment conditions, **(B)** complexity and different pattern types, and **(C)** judgment and pattern type. Error bars represent ±1 *SEM.*

**Figure 4:**
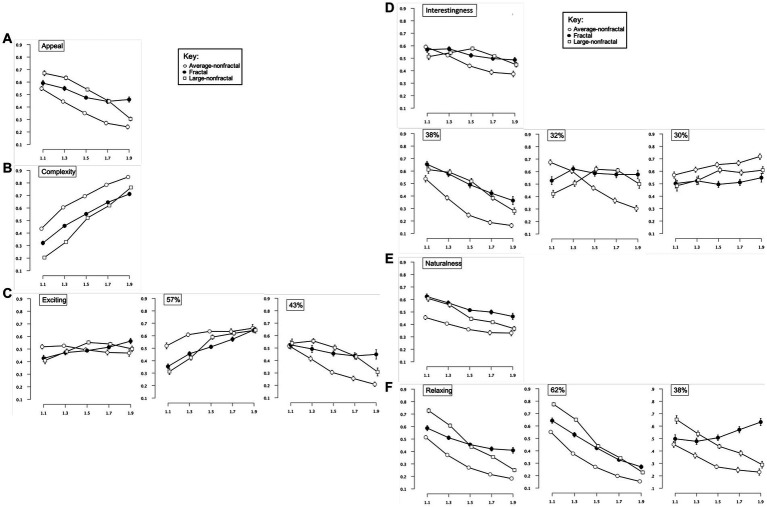
Experiment 1 results for the 3 patterns across 5 different judgment conditions (appeal, complexity, exciting, interestingness, engaging, relaxing). (**A–F** left images) Show plots of mean ratings as a function of pattern complexity (displayed as corresponding fractal dimension “*D*-value”) and pattern type (fractal, Average-nonfractal, Large-nonfractal) for the different judgment conditions (error bars represent standard error of the mean). (**A–F** middle and right images) Show plots of mean ratings as a function of complexity and pattern type for each subpopulation identified with cluster analysis (error bars represent ±1 *SEM*).

A 3-way repeated-measures 3x5x6 ANOVA [Pattern-Type (fractal, Average-nonfractal, Large-nonfractal) × Complexity (equal or matched to *D*-values of 1.1, 1.3, 1.5, 1.7, 1.9) × Judgment (complexity, appeal, naturalness, interest, relaxing, exciting)] was performed using IBM SPSS Statistics for Macintosh (Version 28.0) on rating data for the black-white patterns (recorded as location selected on a rating response slider). Degrees of freedom were corrected for the factors of Pattern-type, Complexity, Judgment, the interaction of Pattern-type and Complexity, Complexity and Judgment, Pattern-type and Judgment, and the three-way interaction of Pattern-type, Complexity, and Judgment using Greenhouse–Geisser estimates of sphericity (ε = 0.826, 0.343, 0.832, 0.338, 0.258, 0.756, and 0.348 respectively). Significant main effects were detected for Complexity [*F*(1.37,127.63) = 17.77, *p* < 0.001, 95% *CI* [0.06,0.27], η_p_^2^ = 0.16], Pattern-type [*F*(1.65,153.72) = 3.78, *p* < 0.001, 95% *CI* [0,0.31], η_p_^2^ = 0.25], and Judgment [*F*(4.16,387.07) = 19.67, *p* < 0.001, 95% *CI* [0.10,0.53], η_p_^2^ = 0.41]. Additional significant interactions were detected between Complexity and Judgment [*F*(5.15,479.28) = 158.30, *p* < 0.001, 95% *CI* [0.58,0.67], η_p_^2^ = 0.63], Complexity and Pattern-type [*F*(2.71,251.69) = 11.37, *p* < 0.001, 95% *CI* [0.04,0.18], η_p_^2^ = 0.10], Pattern-type and Judgment [*F*(7.56,703.03) = 65.43, *p* < 0.001, 95% *CI* [0.36,0.46], η_p_^2^ = 0.41], as well as Complexity, Pattern-type, and Judgment [*F*(13.92,1294.3) = 15.56, *p* < 0.001, 95% *CI* [0.10,0.17], η_p_^2^ = 0.14]. For the Complexity and Judgment interaction, some judgments had ratings that decreased with additional complexity (appeal, interesting, natural, relaxing), while others were relatively flat (exciting) or increased (complexity; Figure 3A). For the Pattern-type and Complexity interaction, ratings for the fractal, Large-nonfractal, and Average-nonfractal patterns were either relatively flat, decreased at the highest level of complexity, or decreased across levels of complexity, respectively (Figure 3B). Finally, for the Judgment by Pattern Type interaction, the differences in ratings among the 3 pattern types varied across judgment type (Figure 3C). The 3-way interaction indicates that the Pattern-type by Complexity interaction varied across Judgment-type, seen more clearly in Figure 4 (leftmost column of graphs). The relationship between the six judgment factors is examined in Table 1, and is followed by a series of planned ANOVA’s and t-tests (with a false-discovery rate (FDR) correction applied to *p*-values in Table 2 to control for family-wise error rates, see Benjamini and Hochberg, 1995 for further details) to better explore the interaction between pattern Complexity and Type across various Judgments and whether these perceptual trends can be better explained by subgroups determined by a 2-step cluster analysis.

**Table 1:** Experiment 1—correlations between the rating categories.

Rating correlations						
	Appeal	Complex	Exciting	Interesting	Natural	Relaxing
Appeal	*-*					
Complex	*r* = 0.02, *p* = 0.85	*-*				
Exciting	*r* = 0.33, *p* = 0.001^*^	*r* = 0.42, *p* < 0.001^**^	*-*			
Interesting	*r* = 0.47, *p* < 0.001^**^	*r* = 0.33, *p* < 0.001^**^	*r* = 0.66, *p* < 0.001^**^	*-*		
Natural	*r* = 0.19, *p* = 0.06	*r* = −0.01, *p* = 0.92	*r* = 0.27, *p* = 0.01^*^	*r* = 0.27, *p* = 0.01^*^	*-*	
Relaxing	*r* = 0.49, *p* < 0.001^**^	*r* = −0.15, *p* = 0.15	*r* = 0.25, *p* = 0.02^*^	*r* = 0.36, *p* < 0.001^**^	*r* = 0.43, *p* < 0.001^**^	*-*

^*^p < 0.05; ^**^p < 0.001.

**Table 2:** Experiment 1—paired samples *t*-Tests between patterns across complexity and judgment.

Paired samples *t*-Tests					
Appeal					
	1.1	1.3	1.5	1.7	1.9
Average nonfractal—fractal	*t* = −1.597	*t* = −4.054	*t* = −5.763	*t* = −7.264	*t* = −8.606
	*p* = 0.13	*p* = 0.01^*^	*p* = 0.004^*^	*p* = 0.003^*^	*p* = 0.002^*^
Average nonfractal—large nonfractal	*t* = −5.699	*t* = −8.333	*t* = −12.709	*t* = −9.645	*t* = −4.319
	*p* = 0.02^*^	*p* = 0.001^*^	*p* = 0.003^*^	*p* = 0.002^*^	*p* = 0.002^*^
Fractal—large nonfractal	*t* = −3.398	*t* = −4.343	*t* = −2.959	*t* = −7.264	*t* = 6.173
	*p* = 0.09	*p* = 0.004^*^	*p* = 0.01^*^	*p* = 0.002^*^	*p* = 0.002^*^
Complex					
	1.1	1.3	1.5	1.7	1.9
Average nonfractal—fractal	*t* = 6.499	*t* = 8.582	*t* = 10.137	*t* = 9.168	*t* = 8.638
	*p* = 0.05^*^	*p* = 0.01^*^	*p* = 0.003^*^	*p* = 0.002^*^	*p* = 0.002^*^
Average nonfractal—large nonfractal	*t* = 19.198	*t* = −3.361	*t* = 12.820	*t* = 12.692	*t* = 7.557
	*p* = 0.01^*^	*p* = 0.01^*^	*p* = 0.003^*^	*p* = 0.002^*^	*p* = 0.002^*^
Fractal—large nonfractal	*t* = 8.102	*t* = −10.454	*t* = 2.150	*t* = 1.564	*t* = −3.119
	*p* = 0.01^*^	*p* = 0.004^*^	*p* = 0.04^*^	*p* = 0.14	*p* = 0.003^*^
Exciting					
	1.1	1.3	1.5	1.7	1.9
Average nonfractal—fractal	*t* = 3.369	*t* = 2.186	*t* = 0.326	*t* = −1.702	*t* = −3.035
	*p* = 0.03^*^	*p* = 0.04^*^	*p* = 0.75	*p* = 0.11	*p* = 0.004^*^
Average nonfractal—large nonfractal	*t* = 4.202	*t* = 1.926	*t* = −2.722	*t* = −2.785	*t* = −1.427
	*p* = 0.01^*^	*p* = 0.07	*p* = 0.01^*^	*p* = 0.01^*^	*p* = 0.18
Fractal—large nonfractal	*t* = 0.710	*t* = −0.409	*t* = −3.129	*t* = −1.154	*t* = 2.225
	*p* = 0.50	*p* = 0.69	*p* = 0.003^*^	*p* = 0.27	*p* = 0.04^*^
Interesting					
	1.1	1.3	1.5	1.7	1.9
Average nonfractal—fractal	*t* = 0.861	*t* = −2.235	*t* = −3.327	*t* = −4.530	*t* = −3.933
	*p* = 0.41	*p* = 0.04^*^	*p* = 0.003^*^	*p* = 0.002^*^	*p* = 0.002^*^
Average nonfractal—large nonfractal	*t* = 2.955	*t* = −0.863	*t* = −6.501	*t* = −6.283	*t* = −3.403
	*p* = 0.01^*^	*p* = 0.41	*p* = 0.003^*^	*p* = 0.002^*^	*p* = 0.002^*^
Fractal—large nonfractal	*t* = 2.409	*t* = 1.336	*t* = −2.577	*t* = −0.977	*t* = 1.567
	*p* = 0.03^*^	*p* = 0.21	*p* = 0.01^*^	*p* = 0.35	*p* = 0.14
Natural					
	1.1	1.3	1.5	1.7	1.9
Average nonfractal—fractal	*t* = −6.675	*t* = −7.801	*t* = −7.908	*t* = −6.637	*t* = −3.942
	*p* = 0.02^*^	*p* = 0.01^*^	*p* = 0.003*	*p* = 0.002^*^	*p* = 0.002^*^
Average nonfractal—large nonfractal	*t* = −8.193	*t* = −6.847	*t* = −4.424	*t* = −4.428	*t* = −2.133
	*p* = 0.01^*^	*p* = 0.005^*^	*p* = 0.003*	*p* = 0.002^*^	*p* = 0.05^*^
Fractal—large nonfractal	*t* = 0.591	*t* = −6.847	*t* = 3.641	*t* = 4.076	*t* = 3.267
	*p* = 0.57	*p* = 0.004^*^	*p* = 0.003^*^	*p* = 0.002^*^	*p* = 0.003^*^
Relaxing					
	1.1	1.3	1.5	1.7	1.9
Average nonfractal—fractal	*t* = −3.364	*t* = −6.619	*t* = −10.337	*t* = −9.572	*t* = −8.991
	*p* = 0.02^*^	*p* = 0.01^*^	*p* = 0.003^*^	*p* = 0.002^*^	*p* = 0.002^*^
Average nonfractal—large nonfractal	*t* = −11.514	*t* = −13.837	*t* = −11.013	*t* = −9.145	*t* = −5.337
	*p* = 0.01^*^	*p* = 0.005^*^	*p* = 0.003^*^	*p* = 0.002^*^	*p* = 0.002^*^
Fractal—large nonfractal	*t* = −6.216	*t* = −5.151	*t* = 0.936	*t* = 3.182	*t* = 6.085
	*p* = 0.01^*^	*p* = 0.004^*^	*p* = 0.04^*^	*p* = 0.003^*^	*p* = 0.002^*^

^*^p < 0.05; ^**^p < 0.001.

##### Appeal

A 2-way 5 × 3 repeated-measures ANOVA [Complexity (equal or matched to *D*-values of 1.1, 1.3, 1.5, 1.7, 1.9) × Pattern-type (fractal, Average-nonfractal, Large-nonfractal)] was completed to examine the impact of Complexity and Pattern-type on ratings of image appeal (Figure 4A). Degrees of freedom were corrected for the factors of Pattern-type, Complexity, and the interaction between Pattern-type and Complexity using Greenhouse–Geisser estimates of sphericity (ε = 0.806, 0.420 and 0.443 respectively). There were significant main effects of Pattern-type [*F*(1.63,149.97) = 63.64, *p* < 0.001, 95% *CI* [0.29,0.50], η_p_^2^ = 0.41] and Complexity [*F*(1.68,156.39) = 113.65, *p* < 0.001, 95% *CI* [0.45,0.62], η_p_^2^ = 0.55], and a significant interaction between Pattern-type and Complexity [*F*(3.54,329.46) = 16.48, *p* < 0.001, 95% *CI* [0.08,0.21], η_p_^2^ = 0.15]. Collapsed over pattern-type, average ratings of appeal ranged from a high of 0.60 (*SD* = 0.18) for the least intricate patterns to a low of 0.33 (*SD* = 0.19) for the most intricate patterns indicating that appeal decreases with additional pattern complexity. Collapsed over complexity, average ratings of appeal were significantly higher for Large-nonfractal (*M* = 0.52; *SD* = 0.08) and fractal (*M* = 0.50; *SD* = 0.12) patterns compared to Average-nonfractal patterns (*M* = 0.37; *SD* = 0.12): Average-nonfractals and fractals [*t*(93) = −7.69, *p* < 0.001, 95% *CI* [−0.17,−0.10], *d* = 0.79]; Average-nonfractals and Large-nonfractals [*t*(93) = −13.31, *p* < 0.001, 95% *CI* [−0.17,−0.13], *d* = 1.37]. These results indicate an overall preference for the fractal and Large-nonfractal patterns, both of which contain large-scale structure. The interaction between Pattern-type and Complexity demonstrates decreasing ratings of appeal across complexity for both the non-fractal patterns but a decrease then leveling off of ratings across complexity for the fractal patterns. Ratings of appeal varied significantly across the three pattern types for individual levels of complexity (Table 2).

To account for possible effects of participant subgroups driving the overall observed trends, a two-step cluster analysis was performed (see Robles et al., 2021). In accordance with and described in Norušis (2012) hierarchical cluster analyses were first completed using Ward’s method to separate individuals into groups using their appeal ratings for each level of pattern complexity. The resulting agglomeration matrix did not indicate a multiple cluster solution, thus not prompting a follow up *k*-means clustering analysis.

##### Complexity

A 2-way 5 × 3 repeated-measures ANOVA [Complexity (equal or matched to *D*-values of 1.1, 1.3, 1.5, 1.7, 1.9) × Pattern-type (fractal, Average-nonfractal, Large-nonfractal)] was completed to examine the impact of Complexity and Pattern-type on pattern complexity judgments (Figure 4B). Degrees of freedom were corrected for the factors of Complexity, Pattern-type, and the interaction between Complexity and Pattern-type using Greenhouse–Geisser estimates of sphericity (ε = 0.472, 0.936 and 0.511 respectively). A significant main effect of Complexity [*F*(1.89,175.41) = 983.37, *p* < 0.001, 95% *CI* [0.89,0.93], η_p_^2^ = 0.91], Pattern-type [*F*(1.87,174.19) = 183.63, *p* < 0.001, 95% *CI* [0.58,0.72], η_p_^2^ = 0.66], and interaction between Complexity and Pattern-type [*F*(4.09,380.12) = 25.88, *p* < 0.001, 95% *CI* [0.14,0.28], η_p_^2^ = 0.22] were identified. Average complexity ratings (collapsed over Pattern-type) ranged from 0.32 (*SD* = 0.27) for the least intricate patterns to 0.77 (*SD* = 0.11) for the most intricate patterns, indicating that participant perception of complexity increased with greater amount of visual complexity. Average complexity ratings (collapsed over Complexity) were highest for Average-nonfractal (*M* = 0.67; *SD* = 0.07), middling for fractal (*M* = 0.54; *SD* = 0.08), and lowest for Large-nonfractal (*M* = 0.49; *SD* = 0.08) patterns, indicating highest overall perceived complexity for the Average-nonfractal patterns, lowest perceived complexity for the Large-nonfractal patterns, with fractal patterns in the middle. The significant differences among the ratings of complexity across the three pattern types were as follows: between Average-nonfractals and fractals [*t*(93) = 12.43, *p* < 0.001, 95% *CI* [0.11,0.16], *d* = 1.28], Average-nonfractals and Large-nonfractals [*t*(93) = 21.40, *p* < 0.001, 95% *CI* [0.17,0.20], *d* = 2.21], and fractals and Large-nonfractals [*t*(93) = 4.83, *p* < 0.001, 95% *CI* [0.03,0.07], *d* = 0.50]. The interaction between Pattern-type and Complexity demonstrates a similar increase in ratings of complexity across complexity for Average-nonfractal and fractal patterns but a steeper increase across complexity for the Large-nonfractal patterns. Significant differences in complexity ratings were identified across the three pattern types for individual levels of complexity (Table 2), however cluster analyses did not indicate a multiple cluster solution.

##### Exciting

A 2-way 5 × 3 repeated-measures ANOVA [Complexity (equal or matched to *D*-values of 1.1, 1.3, 1.5, 1.7, 1.9) × Pattern-type (fractal, Average-nonfractal, Large-nonfractal)] was completed to examine the impact of Complexity and Pattern-type on ratings of pattern excitement (Figure 4C). Degrees of freedom were corrected using Greenhouse–Geisser estimates of sphericity for factors of Complexity, Pattern-type, and their interaction (ε = 0.312, 0.935, and 0.391 respectively). A significant main effect of Complexity [*F*(1.28,119.41) = 3.921, *p* = 0.04, 95% *CI* [0,0.12], η_p_^2^ = 0.04] and interaction between Complexity and Pattern-type [*F*(3.13,290.98) = 10.96, *p* < 0.001, 95% *CI* [04,0.17], η_p_^2^ = 0.11] were identified. Collapsed over Pattern-type, the mean excitement ratings ranged from a low of 0.45 (*SD* = 0.20) for the least intricate patterns to a high of 0.52 (*SD* = 0.15) with moderate-high complexity. The interaction between Pattern-type and Complexity demonstrates different trends for the different pattern types, with an increase in ratings of excitement across complexity for the fractal patterns, an increase then leveling off of ratings for the Large-nonfractal patterns, and flat or slightly decreasing ratings for the Average-nonfractal patterns. Ratings of excitement varied significantly across the three pattern types for individual levels of complexity (Table 2).

A 2-step cluster analysis identified and separated individuals into two subgroups with respect to ratings of pattern excitement (Figure 4C). To test whether the trends found above varied by subgroup, we performed a mixed ANOVA with 5 levels of pattern Complexity, 3 levels of Pattern-type, and 2 Subgroups. Degrees of freedom were corrected using Greenhouse–Geisser estimates of sphericity for factors of Complexity, Pattern-type, and their interaction (ε = 0.412, 0.923 and 0.386, respectively). The main effect of Complexity [*F*(1.65,151.66) = 2.66, *p* = 0.08, 95% *CI* [0,0.09], η_p_^2^ = 0.03] and Pattern-type [*F*(1.85,169.82) = 0.91, *p* = 0.40, 95% *CI* [0,0.05], η_p_^2^ = 0.01]were not significant, but significant interactions were identified between Complexity and Pattern-type [*F*(3.09,284.14) = 11.15, *p* < 0.001, 95% *CI* [0.04,0.17], η_p_^2^ = 0.11], Pattern-type and Cluster membership [*F*(1.85,169.82) = 47.59, *p* < 0.001, 95% *CI* [0.23,0.44], η_p_^2^ = 0.34], Complexity and Cluster membership [*F*(1.65,151.66) = 101.61, *p* < 0.001, 95% *CI* [0.42,0.60], η_p_^2^ = 0.53] as well as a three-way interaction among Complexity, Pattern-type, and Cluster membership [*F*(3.09,284.14) =3.26, *p* = 0.02, 95% *CI* [0,0.08], η_p_^2^ = 0.03]. The larger cluster contains 57% of participants and demonstrates increases in excitement ratings with additional pattern complexity. The smaller cluster encompasses 43% of participants and produces a trend in excitement ratings that decreases with additional complexity, steeply for non-fractal patterns and subtly for fractal patterns. Although these represent opposing trends in judgments of excitement, each group is exemplified by a convergence of peak excitement ratings, respectively at either the lowest or highest levels of pattern complexity. In addition, the Average-nonfractal patterns show the highest and lowest levels of excitement in the larger and smaller subgroups, respectively.

##### Interesting

A 2-way 5 × 3 repeated-measures ANOVA [Complexity (equal or matched to *D*-values of 1.1, 1.3, 1.5, 1.7, 1.9) × Pattern-type (fractal, Average-nonfractal, Large-nonfractal)] examined the impact of Complexity and Pattern-Type on perceived pattern interest (Figure 4D). Degrees of freedom were corrected using Greenhouse–Geisser estimates of sphericity for factors of Complexity, Pattern-type, and their interaction (ε = 0.408, 0.894, and 0.500 respectively). There were significant main effects for Complexity [*F*(1.63,151.61) = 20.36, *p* < 0.001, 95% *CI* [0.08,0.28], η_p_^2^ = 0.18] and Pattern-type [*F*(1.79,166.20) = 10.06, *p* < 0.001, 95% *CI* [0.03,0.18], η_p_^2^ = 0.10], and an interaction between Complexity and Pattern-type [*F*(4,372.16) = 11.67, *p* < 0.001, 95% *CI* [0.05,0.17], η_p_^2^ = 0.11]. Collapsed over Pattern-type, the mean ratings of interest ranged from a high of 0.56 (*SD* = 0.55) for the least intricate patterns to a low of 0.44 (*SD* = 0.23) for the most intricate patterns, suggesting that participants interest decreases with additional pattern complexity. Average interest ratings (collapsed over Complexity) were highest for fractal (*M* = 0.53; *SD* = 0.10) and Large-nonfractal (*M* = 0.52; *SD* = 0.10), and lowest for Average-nonfractal (*M* = 0.46; *SD* = 0.16), indicating greater overall interest for fractal and Large-nonfractal patterns compared to Average-nonfractal patterns: Average-nonfractals and fractals [*t*(93) = −3.59, *p* < 0.001, 95% *CI* [−0.10,−0.03], *d* = 0.37]; Average-nonfractals and Large-nonfractals [*t*(93) = −3.88, *p* < 0.001, 95% *CI* [−0.09,−0.03], *d* = 0.40]. The interaction between Pattern-type and Complexity demonstrates a decrease in interestingness for fractal and Average-nonfractal patterns, with a steeper decrease for the Average-nonfractal patterns, as well as an increase up to mid-complexity patterns then a decrease for higher complexity patterns for the Large-nonfractal patterns. Interestingness ratings varied significantly across the three pattern types for individual levels of complexity (Table 2).

A 2-step cluster analysis identified and separated individuals into three distinct subgroups with respect to ratings of pattern interest (Figure 4D). We performed a mixed ANOVA with 5 levels of pattern Complexity, 3 levels of Pattern-type, and 3 Subgroups. Degrees of freedom were corrected using Greenhouse–Geisser estimates of sphericity for factors of Complexity, Pattern-type, and their interaction (ε = 0.546, 0.881 and 0.564 respectively). Both main effects of Complexity [*F*(2.18,198.72) = 25.932, *p* < 0.001, 95% *CI* [0.12,0.31], η_p_^2^ = 0.22] and Pattern-type [*F*(1.76,160.36) = 10.88, *p* = 0.40, 95% *CI* [0.03,0.20], η_p_^2^ = 0.11] were significant, as well as interactions between Complexity and Pattern-type [*F*(4.51,410.56) = 13.60, *p* < 0.001, 95% *CI* [0.07,0.18], η_p_^2^ = 0.13], Pattern-type and Cluster membership [*F*(3.52,160.36) = 32.29, *p* < 0.001, 95% *CI* [0.29,0.50], η_p_^2^ = 0.42], Complexity and Cluster membership [*F*(4.37,198.72) = 35.46, *p* < 0.001, 95% *CI* [0.33,0.51], η_p_^2^ = 0.44], and a three-way interaction among Complexity, Pattern-type, and Cluster membership [*F*(9.02,410.56) = 9.17, *p* < 0.001, 95% *CI* [0.09,0.22], η_p_^2^ = 0.17]. The first cluster encompassed 38% of participants and produces a trend in which ratings decrease with additional complexity; conversely the third cluster contained 30% of participants and a trend in which ratings increase with additional pattern complexity. In addition, the Average-nonfractal patterns show the lowest and highest levels of interest in the largest and smallest subgroups, respectively. The second cluster was comprised of 32% of participants and represented a general decrease in interest for Average-nonfractal patterns with additional complexity, similar to the largest subgroup, alongside higher ratings for the other two pattern types at higher levels of complexity (complexity levels 1.5 and 1.7 for Average-nonfractal patterns and 1.3–1.9 for fractal patterns).

##### Natural

A 2-way 5 × 3 repeated-measures ANOVA [Complexity (equal or matched to *D*-values of 1.1, 1.3, 1.5, 1.7, 1.9) × Pattern-type (fractal, Average-nonfractal, Large-nonfractal)] assessed the impact of Complexity and Pattern-type on perceived pattern naturalness (Figure 4E). Degrees of freedom were corrected using Greenhouse–Geisser estimates of sphericity for factors of Complexity and interaction between Complexity and Pattern-type (ε = 0.329 and 0.302 respectively). There were significant main effects of pattern Complexity [*F*(1.31,122.25) = 36.43, *p* < 0.001, 95% *CI* [0.15,0.40], η_p_^2^ = 0.28] and Pattern-type [*F*(2,186) = 61.73, *p* < 0.001, 95% *CI* [0.29,0.48], η_p_^2^ = 0.40], and a significant interaction between these factors [*F*(2.42,224.87) = 3.46, *p* = 0.03, 95% *CI* [0,0.45], η_p_^2^ = 0.04]. Collapsed over Pattern-type, the mean ratings of naturalness decreased with additional pattern complexity from a mean of 0.56 (*SD* = 0.19) for the least intricate patterns to a mean of 0.39 (*SD* = 0.23) for most intricate patterns, suggesting that participants’ perception of naturalness decreases with additional pattern complexity. Average naturalness ratings (collapsed over Complexity) were highest for fractal (*M* = 0.54; *SD* = 0.09), middling for Large-nonfractal (*M* = 0.48; *SD* = 0.10), and lowest for Average-nonfractal (*M* = 0.38; *SD* = 0.14), indicating the greatest, middling, and least overall perception of naturalness for fractal, Large-nonfractal, and Average-nonfractal patterns, respectively. The significant differences among the ratings of naturalness across the three pattern types were as follows: between Average-nonfractals and fractals [*t*(93) = −9.85, *p* < 0.001, 95% *CI* [−0.19,−0.13], *d* = 1.02], Average-nonfractals and Large-nonfractals [*t*(93) = −7.21, *p* < 0.001, 95% *CI* [−0.13,−0.07], *d* = 0.74], and fractals and Large-nonfractals [*t*(93) = 4.35, *p* < 0.001, 95% *CI* [0.03,0.08], *d* = 0.45]. The interaction between Pattern-type and Complexity demonstrates a similar decrease in perceived naturalness for fractal and Average-nonfractal patterns, with overall higher ratings of naturalness for the fractal patterns, as well as a steeper decrease in ratings for the Large-nonfractal patterns. Ratings of naturalness showed significant differences across the three pattern types for individual levels of complexity (Table 2), however no significant subgroups were identified for participant ratings of pattern naturalness.

##### Relaxing

A 2-way 5 × 3 repeated-measures ANOVA [Complexity (equal or matched to *D*-values of 1.1, 1.3, 1.5, 1.7, 1.9) × Pattern-type (fractal, Average-nonfractal, Large-nonfractal)] assessed the impact of Complexity and Pattern-type on perceived relaxation (Figure 4F). Degrees of freedom were corrected using Greenhouse–Geisser estimates of sphericity for factors of Complexity, Pattern-type, and their interaction (ε = 0.356, 0.825, and 0.446 respectively). There were significant main effects of pattern Complexity [*F*(1.43,132.49) = 207.80, *p* < 0.001, 95% *CI* [0.60,0.75], η_p_^2^ = 0.69] and Pattern-type [*F*(1.65,153.48) = 108.21, *p* < 0.001, 95% *CI* [0.48,0.65], η_p_^2^ = 0.54] as well as an interaction between these factors [*F*(3.56,331.48) = 26.94, *p* < 0.001, 95% *CI* [0.14,0.29], η_p_^2^ = 0.23]. Collapsed across Pattern-type, average ratings of pattern relaxation ranged from a high of 0.61 (*SD* = 0.16) for the simplest patterns to a low of 0.28 (*SD* = 0.16) for the highest complexity patterns, suggesting that participants perceived patterns as less relaxing with increasing complexity. Collapsed over complexity, average ratings of relaxation were higher for fractal (*M* = 0.48; *SD* = 0.11) and Large-nonfractal (*M* = 0.48; *SD* = 0.09) patterns compared to Average-nonfractal patterns (*M* = 0.31; *SD* = 0.09), indicating an overall greater relaxation response for the fractal and Large-nonfractal patterns, both of which contain large-scale structure: Average-nonfractals and fractals [*t*(93) = −11.28, *p* < 0.001, 95% *CI* [−0.19,−0.14], *d* = 1.16]; Average-nonfractals and Large-nonfractals [*t*(93) = −17.23, *p* < 0.001, 95% *CI* [−0.18,−0.15], *d* = 1.78]. The interaction between Pattern-type and Complexity demonstrates decreasing ratings of relaxation across complexity for both the non-fractal patterns but a decrease then leveling off of ratings across complexity for the fractal patterns. Among the ratings of relaxation significant differences arose across the three pattern types for individual levels of complexity (Table 2).

A 2-step cluster analysis identified and separated individuals into two subgroups with respect to ratings of pattern relaxation. We performed a mixed ANOVA with 5 levels of pattern Complexity, 3 levels of Pattern-type, and 2 Subgroups. Degrees of freedom were corrected using Greenhouse–Geisser estimates of sphericity for factors of Complexity, Pattern-type, and their interaction (ε = 0.459, 0.854, and 0.516, respectively). There were significant main effects for Complexity [*F*(1.84,168.94) = 271.89, *p* < 0.001, 95% *CI* [0.68,0.79], η_p_^2^ = 0.75] and Pattern-type [*F*(1.71,157.16) = 122.57, *p* < 0.001, 95% *CI* [0.47,0.64], η_p_^2^ = 0.57], as well as significant interactions between Complexity and Pattern-type [*F*(4.13,379.78) = 35.28, *p* < 0.001, 95% *CI* [0.20,0.34], η_p_^2^ = 0.28], Complexity and Clusters [*F*(1.84,168.94) = 68.06, *p* < 0.001, 95% *CI* [0.31,0.51], η_p_^2^ = 0.43], Pattern-type and Cluster membership [*F*(1.71,157.16) = 13.71, *p* < 0.001, 95% *CI* [0.04,0.22], η_p_^2^ = 0.13], and a three-way interaction between Complexity, Pattern-type, and Clusters [*F*(4.13,379.78) = 11.99, *p* < 0.001, 95% *CI* [0.06,0.17], η_p_^2^ = 0.12]. The largest cluster encompassed 62% of participants whereas the smaller cluster contained the remaining 38% of individuals. Across both clusters, perceptions of relaxation swiftly decrease with additional complexity for Average-nonfractal and Large-nonfractal patterns. The difference between these clusters is driven by contradictory trends in relaxation ratings for fractal stimuli, with participants in the smaller cluster perceiving higher complexity fractals as more relaxing whereas those in the larger cluster rate lower complexity fractals as more relaxing (Figure 4F).

### Discussion

Experiment 1 explored how a variety of psychological effects are altered by variation in underlying pattern structure. Overall, results indicate that with regards to increasing pattern complexity, viewer perceptions of pattern complexity increase, whereas perceptions of pattern appeal, interestingness, naturalness, and relaxation decrease. Furthermore, perceptions of pattern excitement remain more moderate regardless of pattern complexity. With regard to complexity, the highest overall perceived complexity was for the Average-nonfractal patterns, the middle was for the fractal patterns, and the lowest was for the Large-nonfractal patterns. For the naturalness judgments, the highest overall perceived naturalness was for the fractal patterns, the middle was for the Large-nonfractal patterns, and the lowest was for the Average-nonfractal patterns. In addition, there were overall higher ratings of appeal, relaxation, and interestingness for fractal and Large-nonfractal compared to the matched Average-nonfractal patterns. For appeal and relaxation judgments, Large-nonfractal compared to fractal patterns show a steeper drop off in ratings across levels of complexity. For interesting judgments, peak ratings for Large-nonfractal patterns are for the mid-level of complexity, ratings for fractal patterns are relatively flat, and ratings for original-non fractals decrease with complexity. These results show a distinctive pattern of perceptual responses to the 3 pattern types.

Viewer ratings of pattern excitement and relaxation were shown to be more completely explained through the comparison of 2 subgroups present in the data. The larger subgroups demonstrated lower ratings for relaxation and higher ratings of excitement with the presence of additional pattern complexity. The smaller subgroups demonstrate decreasing ratings across complexity for both non-fractal patterns but a decrease then leveling off of excitement ratings and a flat then increasing relaxation rating across complexity for the fractal patterns. Ratings of pattern interestingness were more completely explained through the comparison of 3 subgroups. The largest group demonstrates decreasing ratings with additional complexity, the smallest subgroup demonstrates the opposite trend, and the middle subgroup demonstrates a more complicated trend in responses. Comprising just over a third of viewers, this group demonstrates an agreement with the largest subgroups’ assessment of Average-nonfractal patterns (i.e., decreasing ratings across levels of complexity), but shows relaxation ratings peaking with moderate-to-high levels of complexity for the Large-nonfractal and fractal patterns. Presented fractal trends in complexity, naturalness, and relaxation ratings reinforce previously established findings (Robles et al., 2021). However, ratings of fractal appeal noticeably deviate from prior findings (Bies et al., 2016a; Abboushi et al., 2019; Robles et al., 2020, 2021) by peaking with lowest pattern complexity and decreasing steadily through moderate-high complexity at which point appeal ratings level off. This deviation is suspected to be a product of context effects created through the mixed presentation of the three pattern types, such that the overall discrepancy in pattern complexity between fractal and nonfractal patterns shifts perceptual tolerance toward the simplest patterns.

## Experiment 2—impact of the incorporation of Euclidean structure on fractal pattern perception

### Materials and methods

#### Stimuli

**Figure 5:**
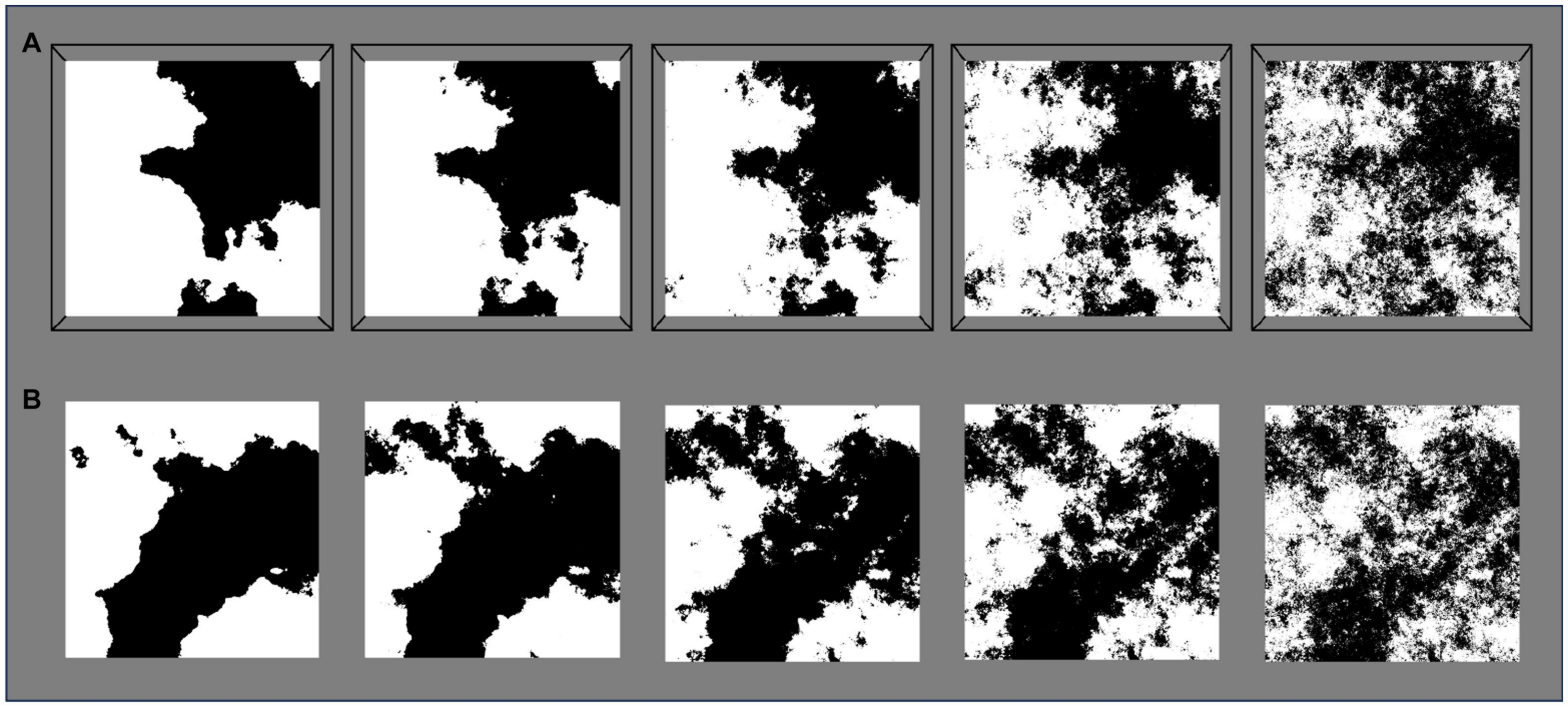
Examples of experiment 2 stimuli. Fractal patterns presented at five different levels of complexity (left–right) with **(A)** simple Euclidean surrounding structure reminiscent of a frame or wall of an interior humanmade space or **(B)** without surrounding boundaries.

To understand how the integration of Euclidean surroundings alters trends in fractal perception, Experiment 2 utilizes a new series of fractal images generated in the same manner as described in Experiment 1. Viewer ratings are compared across trials with stimuli presented in isolation on the computer screen or trials where stimuli are surrounded by a frame composed of one large outer square connected to the pattern through lines connecting the corners of the fractal pattern and the frame (Figure 5). Stimuli consisted of a total of 40 patterns, with 8 examples each of 5 *D*-values (*D* = 1.1, 1.3, 1.5, 1.7. 1.9).

#### Participants

To identify unique changes in perceptual trends due to combining fractal and Euclidean structure, 60 participants were recruited via Prolific,^1^ [November 2022] with the majority of participants (26) residing in the United Kingdom (32 females, age ranging between 18 and 75 years old, mean age 35 years old). Informed consent was acquired following a protocol approved by the Institutional Review Board at the University of Oregon and all participants were compensated with $10 for their time.

#### Visual displays

Experiment 2 was also programmed in PsychoPy3 but presented using the online research study platform of Pavlovia (Peirce et al., 2019). Participants completed the experiment using their personal computers with program stimuli scaled to the individual computer’s respective full-screen dimensions.

#### Design and procedure

Similar to the procedure in Experiment 1, participants rated fractal patterns on 6 different factors (complex, appealing, natural, interesting, relaxing, exciting). Experiment 2 consisted of 12 randomized rating blocks with 20 fractal images ranging in complexity (*D* = 1.1, 1.3, 1.5, 1.7, 1.9), for a total of 40 unique images. Half of the blocks contained fractal images surrounded with a Euclidean frame and the remaining blocks presented images in isolation. Each block consisted of a singular judgment type and an on-screen slider located beneath each image was used to record self-report ratings for each pattern. The rating task was completed in the manner as described in Experiment 1.

### Results

Data from 40 participants were retained from the 60 individuals who participated in the experiment. Data were excluded due to (a) failure to complete the study (8), (b) failure to follow instructions (8), or (c) if in at least 3 blocks participants recorded the same rating for greater than four consecutive trials (4).

#### Pattern rating task

**Figure 6:**
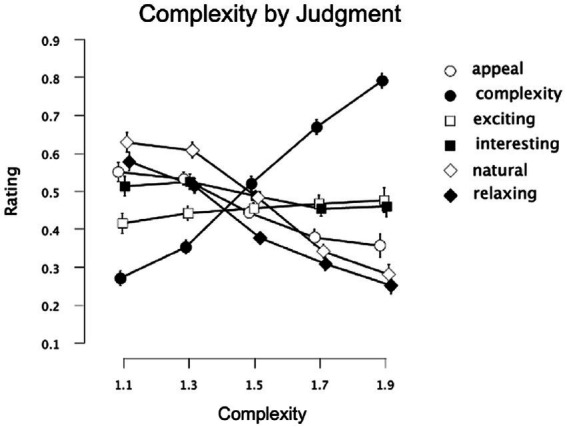
Experiment 2 results for unipolar ratings of fractal patterns varying in surrounding context. Results show a significant interaction among two of the experiment’s factors: pattern complexity (presented in fractal dimension “*D*-value”) and judgment type (appeal, complexity, exciting, interestingness, naturalness, relaxing). Participant rating (on a scale from 0–1) is plotted as a function of *D*-value and different judgment conditions. Error bars represent ±1 *SEM.*

Similar to Experiment 1, a 3-way repeated-measures 2 × 5 × 6 ANOVA [Context (Euclidean-frame, no-frame) × Complexity (*D*-values of 1.1, 1.3, 1.5, 1.7, 1.9) × Judgment (complexity, exciting, appeal, interesting, natural, relaxing)] was performed on rating data for the fractal patterns. Degrees of freedom were corrected using Greenhouse–Geisser estimates of sphericity for factors of Complexity, Judgment, the interactions between Context and Complexity, Complexity and Judgment, Context and Judgment, and the three-way interaction between Context, Complexity, and Judgment (ε = 0.295, 0.612, 0.510, 0.213, 0.750, and 0.420 respectively). Significant main effects were identified for Judgment [*F*(3.06,119.43) = 9.29, *p* < 0.001, 95% *CI* [0.07,0.30], η_p_^2^ = 0.19] and a significant interaction between Complexity and Judgment [*F*(4.27,166.50) = 38.24, *p* < 0.001, 95% *CI* [0.38,0.57], η_p_^2^ = 0.50]. Coinciding with findings from Experiment 1, the Complexity and Judgment interaction is demonstrated through ratings that decreased in the presence of additional complexity (appeal, interesting, natural, relaxing), while others were relatively flat (exciting) or increased (complexity; Figure 6). The relationship between the six judgment factors is examined in Table 3, and a series of planned ANOVA’s and t-tests follow (with FDR correction applied to p-values in Table 4 to control for family-wise error rates) to determine whether observed perceptual trends can be better explained by subgroups determined by a 2-step cluster analysis.

**Table 3:** Experiment 2—correlations between the rating categories.

Rating correlations						
	Appeal	Complex	Exciting	Interesting	Natural	Relaxing
Appeal	*-*					
Complex	*r* = −0.13, *p* = 0.42	*-*				
Exciting	*r* = 0.74, *p* < 0.001^**^	*r* = −0.02, *p* = 0.90	*-*			
Interesting	*r* = 0.74, *p* < 0.001^**^	*r* = −0.11, *p* = 0.51	*r* = 0.75, *p* < 0.001^**^	*-*		
Natural	*r* = 0.51, *p* < 0.001^**^	*r* = −0.34, *p* = 0.03^*^	*r* = 0.53, *p* < 0.001^**^	*r* = 0.64, *p* < 0.001^**^	*-*	
Relaxing	*r* = 0.58, *p* < 0.001^**^	*r* = −0.33, *p* = 0.04^*^	*r* = −0.66, *p* < 0.001^**^	*r* = 0.65, *p* < 0.001^**^	*r* = 0.64, *p* < 0.001^**^	*-*

^*^p < 0.05; ^**^p < 0.001.

**Table 4:** Experiment 2—paired samples *t*-Tests between patterns across complexity and judgment.

Paired Samples *t*-Tests					
	1.1	1.3	1.5	1.7	1.9
Appeal: Euclidean frame—no frame	*t* = −0.73	*t* = 0.11	*t* = −0.60	*t* = 0.74	*t* = 0.62
	*p* = 0.71	*p* = 0.94	*p* = 0.72	*p* = 0.73	*p* = 0.74
Complex: Euclidean frame—no frame	*t* = 2.35	*t* = 1.68	*t* = 1.22	*t* = 0.62	*t* = −1.24
	*p* = 0.30	*p* = 0.43	*p* = 0.49	*p* = 0.77	*p* = 0.51
Exciting: Euclidean frame—no frame	*t* = 1.99	*t* = 1.32	*t* = 0.29	*t* = −1.33	*t* = −2.03
	*p* = 0.45	*p* = 0.57	*p* = 0.90	*p* = 0.52	*p* = 0.38
Interesting: Euclidean frame—no frame	*t* = 1.59	*t* = 1.32	*t* = 2.51	*t* = 1.97	*t* = 1.54
	*p* = 0.50	*p* = 0.50	*p* = 0.60	*p* = 0.36	*p* = 0.43
Natural: Euclidean frame—no frame	*t* = 0.55	*t* = 1.18	*t* = 0.97	*t* = 0.13	*t* = 0.12
	*p* = 0.73	*p* = 0.50	*p* = 0.60	*p* = 0.99	*p* = 0.98
Relaxing: Euclidean frame—no frame	*t* = 0.81	*t* = 0.08	*t* = 1.77	*t* = −0.50	*t* = −1.0
	*p* = 0.70	*p* = 0.93	*p* = 0.40	*p* = 0.74	*p* = 0.62

^*^p < 0.05; ^**^p < 0.001.

##### Appeal

**Figure 7:**
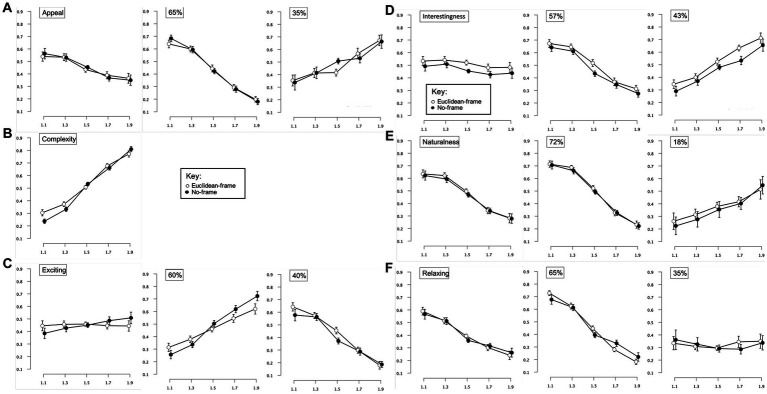
Experiment 2 results for fractal patterns with variation in surrounding context (Euclidean-frame, no-frame) for 6 different judgment conditions (appeal, complexity, exciting, interestingness, naturalness, relaxing). (**A–F** left images) Show plots of mean ratings as a function of fractal dimension (*D*-value) and 2 context conditions (Euclidean-frame, no-frame) for the different judgment conditions (error bars represent standard error of the mean). (**A–F** middle and right images) Show plots of mean ratings as a function of fractal dimension (*D*-value) and 2 context conditions (Euclidean-frame, no-frame) for each subpopulation identified with cluster analysis (error bars represent ±1 *SEM*).

A 2-way 2 × 5 repeated-measures ANOVA [Context (Euclidean-frame, no-frame) × Complexity (*D*-values of 1.1, 1.3, 1.5, 1.7, 1.9)] was completed to examine the impact of surrounding context and pattern complexity on ratings of image appeal (Figure 7A). Degrees of freedom were corrected using Greenhouse–Geisser estimates of sphericity for the factors of Complexity and interaction between Context and Complexity (ε = 0.295 and 0.518 respectively). A significant main effect was identified for Complexity [*F*(1.18,46) = 8.74, *p* = 0.003, 95% *CI* [0.02,0.36], η_p_^2^ = 0.18]. Average ratings of appeal ranged from a high of 0.55 (*SD* = 0.24) for the least intricate patterns to a low of 0.36 (*SD* = 0.27) for the most intricate patterns suggesting that appeal decreases with additional pattern complexity. Paired sample *t*-tests revealed no significant differences in appeal between patterns that vary in surrounding context.

In accordance with the subgroup analysis used in Experiment 1, a two-step cluster analysis was performed to determine the impact of possible subgroups on overall trends (first using hierarchical cluster analyses with Ward’s method to separate individuals into groups then follow up with *k*-means clustering analysis for the number of indicated groups). This 2-step clustering method indicated the presence of two clusters in the data. We performed a mixed ANOVA with 5 levels of pattern Complexity, 2 levels of Context, and 2 Subgroups. Degrees of freedom were corrected for the factors of Complexity and interaction of Complexity and Context using Greenhouse–Geisser estimates of sphericity (ε = 0.418 and 0.499 respectively). Despite the main effect of context not being significant [*F*(1,38) = 0.01, *p* = 0.93, 95% *CI* [0,0.01], η_p_^2^ = 0.00], Complexity [*F*(1.67,63.48) = 5.54, *p* = 0.009, 95% *CI* [0.01,0.27], η_p_^2^ = 0.13] and the interaction between Complexity and Subgroup [*F*(1.67,63.48) = 84.15, *p* < 0.001, 95% *CI* [0.55,0.76], η_p_^2^ = 0.69] were significant. The larger subgroup (65% of participants) demonstrates ratings that steeply decrease with additional pattern complexity, while smaller subgroup (35% of participants) produces a trend in ratings that increase with increasing complexity. These findings support previous individual differences research identifying opposing trends in judgments of pattern appeal (Spehar et al., 2016; Street et al., 2016; Robles et al., 2021).

##### Complexity

A 2-way 2 × 5 repeated-measures ANOVA [Context (Euclidean-frame, no-frame) x Complexity (*D*-values of 1.1, 1.3, 1.5, 1.7, 1.9)] was completed to examine the impact of surrounding Context and pattern Complexity on complexity judgments (Figure 7B). Degrees of freedom were corrected for the factors of Complexity and the interaction of Complexity and Context using Greenhouse–Geisser estimates of sphericity (ε = 0.522 and 0.452 respectively). The only significant effect was identified for Complexity [*F*(2.09,81.45) = 226.48, *p* < 0.001, 95% *CI* [0.79,0.89], η_p_^2^ = 0.85]. Average complexity ratings ranged from 0.27 (*SD* = 0.14) for the least intricate patterns to 0.83 (*SD* = 0.15) for the most intricate patterns, indicating that perceptions of complexity increased with greater amount of visual complexity. Paired samples *t*-tests revealed no significant differences in perceived complexity for patterns with or without surrounding context (Table 4).

##### Exciting

A 2-way 2 × 5 repeated-measures ANOVA [Context (Euclidean-frame, no-frame) x Complexity (*D*-values of 1.1, 1.3, 1.5, 1.7, 1.9)] was completed to examine the impact of Context and Complexity on ratings of pattern excitement (Figure 7C). Degrees of freedom were corrected for the factors of Complexity and the interaction of Complexity and Context using Greenhouse–Geisser estimates of sphericity (ε = 0.308 and 0.437, respectively). No significant main effects were identified (Complexity [*F*(1.23,48.07) = 0.57, *p* = 0.57, 95% *CI* [0,0.13], η_p_^2^ = 0.01]; Context (1,39) = 0.01, *p* = 0.77, 95% *CI* [0,0.01], η_p_^2^ = 0.01). However, a significant interaction exists between Complexity and Pattern-type [*F*(1.75,68.16) = 3.58, *p* = 0.07, 95% *CI* [0,0.21], η_p_^2^ = 0.08]. Across complexity, ratings for excitement were flat for the Euclidean-frame patterns but increasing for the No-frame patterns. Paired samples *t*-tests revealed no significant differences in perceived excitement for patterns with or without surrounding context (Table 4).

A 2-step cluster analysis identified and separated individuals into two subgroups with respect to ratings of pattern excitement (Figure 7C). A mixed ANOVA was performed with 5 levels of pattern Complexity, 2 levels of Context, and 2 Subgroups. Degrees of freedom were corrected for the factors of Complexity and the interaction of Complexity and Pattern-type using Greenhouse–Geisser estimates of sphericity (ε = 0.524 and 0.435 respectively). The main effects of Complexity [*F*(2.10,79.59) = 0.57, *p* = 0.57, 95% *CI* [0,0.08], η_p_^2^ = 0.02] and Context [*F*(1,38) = 0.09, *p* = 0.77, 95% *CI* [0,0.09], η_p_^2^ = 0.00] were not significant. The only significant interaction was identified between Complexity and Subgroup [*F*(2.10,79.59) = 93.12, *p* < 0.001, 95% *CI* [0.59,0.77], η_p_^2^ = 0.71]. The remaining interactions [Complexity and Context (*F*(1.74,66.11) = 2.94, *p* = 0.07, 95% *CI* [0,0.20], η_p_^2^ = 0.07)], Context and Subgroup [*F*(1,38) = 3.51, *p* = 0.07, 95% *CI* [0,0.27], η_p_^2^ = 0.09], Complexity, Context, and Subgroup [*F*(1.74,66.11) = 1.44, *p* = 0.24, 95% *CI* [0,0.14], η_p_^2^ = 0.04] were not significant. The larger subgroup (60% of participants) produced an increasing trend in ratings with additional pattern complexity, whereas conversely, the smaller subgroup (40% of participants) produced a decreasing trend with additional complexity.

##### Interesting

A 2-way 2 × 5 repeated-measures ANOVA [Context (Euclidean-frame, no-frame) x Complexity (*D*-values of 1.1, 1.3, 1.5, 1.7, 1.9)] examined the impact of Complexity and Context on perceived pattern interest (Figure 7D). Degrees of freedom were corrected for the factors of Complexity and the interaction of Complexity and Context using Greenhouse–Geisser estimates of sphericity (ε = 0.297 and 0.645 respectively). The sole significant effect was found for Context [*F*(1,39) = 10.64, *p* = 0.002, 95% *CI* [0.03,0.41], η_p_^2^ = 0.21]. There were no significant effects for Complexity [*F*(1.12,46.33) = 1.15, *p* = 0.30, 95% *CI* [0,0.16], η_p_^2^ = 0.03] or the interaction between Complexity and Context [*F*(2.58,100.68) = 0.27, *p* = 0.82, 95% *CI* [0,0.04], η_p_^2^ = 0.01]. Paired samples *t*-tests revealed no significant differences in perceived interest for patterns with or without surrounding context (Table 4). Overall, patterns surrounded by additional Euclidean context were rated more interesting than those without.

A 2-step cluster analysis identified and separated individuals into two distinct subgroups with respect to ratings of pattern interest (Figure 7D). We performed a mixed ANOVA with 5 levels of pattern Complexity, 3 levels of Context, and 2 Subgroups. Degrees of freedom were corrected for the factors of Complexity and the interaction of Complexity and Pattern-type with Greenhouse–Geisser estimates of sphericity (ε = 0.435 and 0.635 respectively). The main effect of Context [*F*(1,38) = 10.66, *p* = 0.002, 95% *CI* [0.03,0.42], η_p_^2^ = 0.22] and interaction between Complexity and Subgroup [*F*(1.74,66.18) = 82.19, *p* < 0.001, 95% *CI* [0.54,0.76], η_p_^2^ = 0.68] were significant. However Complexity [*F*(1.74,66.18) = 0.58, *p* = 0.54, 95% *CI* [0,0.10], η_p_^2^ = 0.02] and interactions between Complexity and Context [*F*(2.54,96.45) = 0.25, *p* = 0.83, 95% *CI* [0,0.04], η_p_^2^ = 0.01], Context and Subgroup [*F*(1,38) = 0.23, *p* = 0.63, 95% *CI* [0,0.13], η_p_^2^ = 0.01] and three-way interaction between Complexity, Context, and Subgroup [*F*(2.54,96.45) = 0.69, *p* = 0.54, 95% *CI* [0,0.08], η_p_^2^ = 0.02] were not. The larger subgroup (57% of participants) demonstrates a general decrease in interest for patterns of additional complexity whereas the smaller subgroup (43% of participants) produces a trend that linearly increases with additional complexity.

##### Natural

A 2-way 2 × 5 repeated-measures ANOVA [Context (Euclidean-frame, no-frame) x Complexity (*D*-values of 1.1, 1.3, 1.5, 1.7, 1.9)] assessed the impact of Complexity and Context on perceived pattern naturalness (Figure 7E). Degrees of freedom were corrected for the factors of Complexity and the interaction of Complexity and Context using Greenhouse–Geisser estimates of sphericity (ε = 0.326 and 0.804 respectively). The main effect of pattern Complexity [*F*(11.01,50.90) = 33.36, *p* < 0.001, 95% *CI* [0.77,0.89], η_p_^2^ = 0.46] was significant, whereas Context [*F*(1,39) = 0.94, *p* = 0.34, 95% *CI* [0,0.17], η_p_^2^ = 0.02] and the interaction between Complexity and Context [*F*(3.22,125.49) = 0.32, *p* = 0.83, 95% *CI* [0,0.04], η_p_^2^ = 0.01] were not. Overall trends demonstrate decreased perceptions of naturalness for patterns of greater complexity with average ratings of 0.63 (*SD* = 0.25) for patterns with *D*-value = 1.1 and 0.28 (*SD* = 0.22) for *D*-value = 1.9, with no significant differences between patterns varying in Context at the 5 levels of complexity.

A 2-step cluster analysis identified and separated individuals into two subgroups with respect to ratings of pattern naturalness. We performed a mixed ANOVA with 5 levels of pattern Complexity, 3 levels of Context, and 2 Subgroups. Degrees of freedom were corrected for the factors of Complexity and the interaction of Complexity and Context using Greenhouse–Geisser estimates of sphericity (ε = 0.433 and 0.804 respectively). The main effect of Complexity [*F*(1.73,65.85) = 4.71, *p* = 0.02, 95% *CI* [0,0.25], η_p_^2^ = 0.11] and the interaction of Complexity and Subgroup [*F*(1.73,65.85) = 41.40, *p* < 0.001, 95% *CI* [0.34,0.63], η_p_^2^ = 0.52] were found to be significant while Context [*F*(1,38) = 0.54, *p* = 0.47, 95% *CI* [0,0.15], η_p_^2^ = 0.01], interactions between Complexity and Context [*F*(3.22,122.23) = 0.34, *p* = 0.81, 95% *CI* [0,0.04], η_p_^2^ = 0.01], Context and Subgroup [*F*(1,38) = 0, *p* = 0.99, 95% *CI* [0,0], η_p_^2^ = 0.00], and Complexity, Context, and Subgroup [*F*(3.22,122.23) = 0.22, *p* = 0.89, 95% *CI* [0,0.03], η_p_^2^ = 0.01] were not significant. Ratings from the large subgroup (72% of individuals) indicated a steep decrease in perceptions of pattern naturalness with additional complexity, whereas those of the small subgroup (18% of participants) indicated a more subtle increase in perceptions of pattern naturalness with additional complexity.

##### Relaxing

A 2-way 2 × 5 repeated-measures ANOVA [Context (Euclidean-frame, no-frame) x Complexity (*D*-values of 1.1, 1.3, 1.5, 1.7, 1.9)] addressed the impact of Complexity and Context on perceived pattern relaxation (Figure 7F). Degrees of freedom were corrected for the factors of Complexity, Pattern-type, and the interaction between the two using Greenhouse–Geisser estimates of sphericity (ε = 0.343 and 0.653 respectively). The only significant effect identified was that of pattern Complexity [*F*(1.37,53.48) = 27.66, *p* < 0.001, 95% *CI* [0.21,0.56], η_p_^2^ = 0.42]. Effects of Context [*F*(1,39) = 0.23, *p* = 0.63, 95% *CI* [0,0.12], η_p_^2^ = 0.01] and the interaction between Complexity and Context [*F*(2.61,101.93) = 1.01, *p* = 0.26, 95% *CI* [0,0.09], η_p_^2^ = 0.03] were not significant (see Table 4). Collapsed across Context, average ratings of pattern relaxation ranged from a high of 0.58 (*SD* = 0.24) for patterns with a *D*-value = 1.1 to a low of 0.25 (*SD* = 0.21) with patterns possessing a *D*-value = 1.9, suggesting that participants perceived patterns as less relaxing with increasing complexity.

A 2-step cluster analysis identified and separated individuals into two subgroups with respect to ratings of pattern relaxation. We performed a mixed ANOVA with 5 levels of pattern Complexity, 3 levels of Context, and 2 Subgroups. Degrees of freedom were corrected for the factors of Complexity and the interaction of Complexity and Context using Greenhouse–Geisser estimates of sphericity (ε = 0.413 and 0.655 respectively). Significant effects were identified for Complexity [*F*(1.65,62.81) = 23.52, *p* < 0.001, 95% *CI* [0.19,0.52], η_p_^2^ = 0.38] and the interaction between Complexity and Subgroup [*F*(1.65,62.81) = 22.48, *p* < 0.001, 95% *CI* [0.18,0.51], η_p_^2^ = 0.37]. The effects of Context [*F*(1,38) = 0.22 *p* = 0.64, 95% *CI* [0,0.13], η_p_^2^ = 0.01], and interactions between Complexity and Context [*F*(2.61,99.61) = 0.43, *p* = 0.70, 95% *CI* [0,0.63], η_p_^2^ = 0.01], Context and Context, and Subgroup [*F*(2.61,99.61) = 2.70, *p* = 0.06, 95% *CI* [0,0.16], η_p_^2^ = 0.07] were not significant. The difference between the subgroups is driven by contradictory response patterns in relaxation ratings for fractal patterns, with participants in the larger subgroup (65% of participants) perceiving lower complexity fractals as highly relaxing, with ratings of relaxation steeply decreasing with the presence of additional complexity. In contrast, those in the smaller subgroup (35% of participants) remain more moderate in their ratings of pattern relaxation across pattern complexity (Figure 7F).

### Discussion

Experiment 2 maintains the same methodological structure and perceptual decisions as described in Experiment 1 but employs only fractal patterns either presented alone or in the presence of a surrounding Euclidean frame (referred to as Euclidean context). In alignment with findings from Experiment 1, judgments of complexity increased with additional *D*-value whereas ratings of appeal, naturalness, and relaxation decreased. Similarly, perceptions of pattern interest and excitement remained more moderate regardless of pattern complexity with interest and excitement showing slightly negative or positive relationships with *D-*value, respectively. Overall, perceptual judgments of pattern excitement and interestingness varied depending on the presence of surrounding Euclidean context, such that patterns with a frame were rated more interesting overall, and maintained flatter ratings of perceived excitement in comparison to fractal patterns without surrounding context which increased with additional pattern complexity. There were 2 subgroups in all judgment conditions aside from pattern complexity. In 4 judgments (pattern appeal, excitement, interestingness, and naturalness) the smaller subgroup trend is in the opposite direction of that of the larger subgroup, while for ratings of pattern relaxation the smaller subgroup deviates from the overall trend with moderate ratings of relaxation across all levels of complexity.

## General discussion

Since modern society’s shift away from the previously agrarian lifestyle, humans have begun spending the majority of their time indoors resulting in a new series of health consequences propelled by the additional exertion of energy required to process non-natural spatial frequencies (Hagerhall et al., 2008; O’Hare and Hibbard, 2011; Penacchio and Wilkins, 2015; Le et al., 2017; Ogawa and Motoyoshi, 2020). In an effort to minimize the higher rates of visual strain, headaches, and general stress associated with Euclidean surroundings, research has sought to integrate natural arrangements into human-made spaces through the inclusion of fractal displays (Taylor and Sprott, 2008; Abboushi et al., 2019; Robles et al., 2021; Smith et al., 2020). Driven by the apparent ease with which the visual system is able to process these patterns (Hagerhall et al., 2008; Taylor and Spehar, 2016; Taylor et al., 2018), introduction of fractal designs into already existing structures can alter viewer perceptions of the space without impacting the function of the space as a whole (Taylor et al., 2005; Hagerhall et al., 2015). To better inform selection of effective installments, it is critical to understand how pattern characteristics alter viewer perception both in isolation and in non-natural spaces.

Across two experiments, the current study serves as the first research to isolate the unique contribution of fractal structure on visual perception as a whole and provides findings as to how these perceptions act in the presence of the simplest prototypical Euclidean context. Both studies demonstrate similar trends in viewer experience of visual patterns despite variations in complexity, underlying structure, and context. Experiment 1 used 3 different types of stimuli (fractal, Average-nonfractal, Large-nonfractal) to demonstrate how variations in pattern structure produce shifts in viewer perception driven by the pattern’s complexity. Perceptions of pattern complexity provide insight into driving factors behind the 5 additional judgments. Although two patterns shared a lack of fractal structure and statistical complexity, they are viewed as significantly different in complexity, with the Average-nonfractal pattern being perceived as the most complex at every level of generated complexity compared to the Large-nonfractal pattern being typically seen as lowest in complexity, and fractal patterns falling somewhere between. The mixed presentation of these patterns, which are broadly different in complexity, likely factors into the remaining observed trends. Akin to how the presence of symmetry and exactness of pattern repetition found in “exact” fractal patterns can increase a viewer’s tolerance of pattern complexity (Bies et al., 2016a; Robles et al., 2021), the presence of overwhelming complexity in Average-nonfractals has the capability to cement viewer inclination toward simplicity.

The results from Experiment 1 suggest that overall (1) there are greater positive responses (higher ratings of appeal, relaxation, and interestingness) to fractal compared to size-matched non-fractal control stimuli (i.e., the Average-nonfractal patterns), (2) these positive responses occur when large-scale structure is present (i.e., for fractal and Large-nonfractal patterns), and (3) removing fractal structure while retaining mean size information (Average-nonfractal patterns) not only increases the perception of complexity but simultaneously reduces the perceived naturalness of the patterns. The finding of generally positive responses to fractal and Large-nonfractal patterns points to the important role of large-scale structure that is present in both of these pattern types. There are also interesting differences in perceptual responses between these pattern types. The fractal compared to the Large-nonfractal patterns are perceived as both more complex and more natural. Interest peaks at lower levels of complexity for the fractal patterns and mid-level complexity for the Large-nonfractal patterns, while excitement peaks at the highest levels of complexity for the fractal patterns and mid-level complexity for the Large-nonfractal patterns. Finally, perceived pattern appeal and relaxation for the Large-nonfractal compared to fractal patterns starts higher, but drops off more rapidly with increasing complexity. Fractals are perceived as distinctly different than nonfractal arrangements, exemplified in ratings of higher complexity patterns where fractals are consistently associated with greater appeal, naturalness, relaxation, and excitement than even Large-nonfractal arrangements despite similarities in perceived complexity and interestingness. These differences among the perceptions of fractal and matched non-fractal patterns provide strong evidence for human sensitivity to fractal structure that characterizes natural objects and environments.

Whereas Experiment 1 serves to establish the role of complexity and pattern structure to predictions of viewer experience, Experiment 2 functions to apply these considerations more directly to the fundamental task of incorporating fractal patterns into Euclidean space (i.e., the built environment). Although fractal patterns may produce stable trends when presented in isolation on a screen, it is imperative to confirm their expected effects when they are embedded in contrasting unnatural human-made design. By comparing fractal perceptions with and without the incorporation of a rudimentary Euclidean context (i.e., a Euclidean frame), results reinforce the perceptual trends established in Experiment 1 irrespective of the inclusion of Euclidean context. Specifically, perceptions of pattern appeal, interestingness, naturalness, and relaxation decrease with increasing *D*-value, while ratings of complexity increase, and those of interest remain more moderate across all levels of complexity. Driven by the imposed pattern boundary created by the Euclidean frame, the effects of nonfractal structure produce experiences of greater interest, and unchanging judgments of excitement across complexity compared to context-free fractals in which ratings of excitement increase with increases in complexity. Thus, despite robust perceptions of patterns across perturbations of structure and surrounding context, selection of optimal patterns for occupant wellbeing must account for the interaction of general pattern complexity with regards to its intended environment.

Viewer subgroupings have a significant impact on overall trends, further substantiating previous findings of individual differences in preference for fractal complexity (Spehar et al., 2016; Street et al., 2016; Bies et al., 2016a; Robles et al., 2021). Opposing subpopulation trends are found for all perceptual ratings aside from complexity which serves to inform design choices by emphasizing the consideration of perceived installment complexity (quantified with *D*-value in the case of fractal patterns) to align with the highest rates of desired experience for the greatest number of viewers.

Lastly, the similarity of the general trends reported between the two studies highlights the seemingly universal nature of fractal perception. While not immense, the expansion in participant age, ethnicity, national origin, natural surroundings, as well as presentation format (in lab or on-line survey) did not seem to impact shared perceptions. This result suggests that perceptions of fractal patterns are unlikely to be altered by experiences of more diverse biomes and thus promotes the application of natural patterns in a broad range of locations. Although this study recruits from both a convenient population as well as a broader group of cultures and countries, our findings are still limited due to an overarching homogeneity of “WEIRD” participant samples. Holistically, findings from Experiment 1 emphasizes the significance of pattern complexity and structure on fractal perception whereas Experiment 2 reinforces the importance of considering Gestalt aspects of complexity when incorporating fractal patterns into surrounding occupant space. The current study serves to provide a foundational understanding of how pattern structure impacts integration with built environments and encourages future work to explore how responses to fractal structure interact with more prominent Euclidean context (through more extended 2D displays, extension to 3D using Virtual Reality technology, as well as with physical installations) and can be optimized for specific categories of locations and products to maximize occupant benefit.

## Data availability statement

The datasets presented in this study can be found in online repositories. The names of the repository/repositories and accession number(s) can be found in the article/supplementary material.

## Ethics statement

The studies involving human participants were reviewed and approved by University of Oregon Research Compliance Services. The patients/participants provided their written informed consent to participate in this study.

## Author contributions

KR, NG-H, RT, and MS contributed to the study design. KR and NG-H contributed to stimulus generation. KR contributed to programming the experiments and testing and data collection. KR and MS contributed to the data analysis and interpretation and drafted the manuscript. All authors contributed to the article and approved the submitted version.

## Conflict of interest

The authors declare that the research was conducted in the absence of any commercial or financial relationships that could be construed as a potential conflict of interest.

## Publisher’s note

All claims expressed in this article are solely those of the authors and do not necessarily represent those of their affiliated organizations, or those of the publisher, the editors and the reviewers. Any product that may be evaluated in this article, or claim that may be made by its manufacturer, is not guaranteed or endorsed by the publisher.

## Footnotes

^1^ http://www.prolific.co/
